# Enhancement of the Start-Up Time for Microliter-Scale Microbial Fuel Cells (µMFCs) via the Surface Modification of Gold Electrodes

**DOI:** 10.3390/mi11070703

**Published:** 2020-07-21

**Authors:** Begüm Şen-Doğan, Meltem Okan, Nilüfer Afşar-Erkal, Ebru Özgür, Özge Zorlu, Haluk Külah

**Affiliations:** 1 Department of Micro and Nanotechnology, Middle East Technical University, Ankara 06800, Turkey; begum.sen@metu.edu.tr (B.Ş.-D.); maydin@mems.metu.edu.tr (M.O.); 2 METU MEMS Research and Application Center, Ankara 06800, Turkey; ebru.ozgur@mikrobiyo.com.tr (E.Ö.); ozge.zorlu@mikrobiyo.com.tr (Ö.Z.); 3 Mikro Biyosistemler A.S., Ankara 06530, Turkey; nilufer.erkal@mikrobiyo.com.tr; 4 Department of Electrical and Electronics Engineering, Middle East Technical University, Ankara 06800, Turkey

**Keywords:** microbial fuel cell, biofilm, surface modification, thiol, *Shewanella oneidensis*, MEMS

## Abstract

Microbial Fuel Cells (MFCs) are biological fuel cells based on the oxidation of fuels by electrogenic bacteria to generate an electric current in electrochemical cells. There are several methods that can be employed to improve their performance. In this study, the effects of gold surface modification with different thiol molecules were investigated for their implementation as anode electrodes in micro-scale MFCs (µMFCs). Several double-chamber µMFCs with 10.4 µL anode and cathode chambers were fabricated using silicon-microelectromechanical systems (MEMS) fabrication technology. µMFC systems assembled with modified gold anodes were operated under anaerobic conditions with the continuous feeding of anolyte and catholyte to compare the effect of different thiol molecules on the biofilm formation of *Shewanella oneidensis* MR-1. Performances were evaluated using polarization curves, Electrochemical Impedance Spectroscopy (EIS), and Scanning Electron Microcopy (SEM). The results showed that µMFCs modified with thiol self-assembled monolayers (SAMs) (cysteamine and 11-MUA) resulted in more than a 50% reduction in start-up times due to better bacterial attachment on the anode surface. Both 11-MUA and cysteamine modifications resulted in dense biofilms, as observed in SEM images. The power output was found to be similar in cysteamine-modified and bare gold µMFCs. The power and current densities obtained in this study were comparable to those reported in similar studies in the literature.

## 1. Introduction

**Figure 1:**
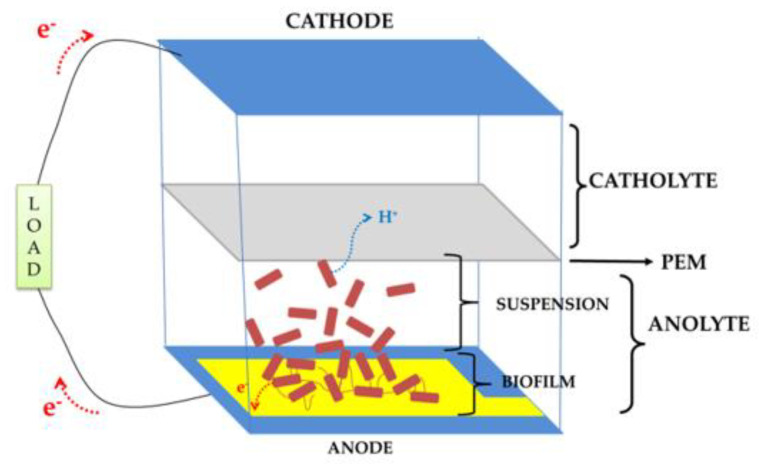
A double-chamber bioanode Microbial Fuel Cell (MFC) schematic.

Microbial Fuel Cells (MFCs) are bioreactors converting the energy stored in chemical bonds of organic compounds (e.g., glucose, lactate, etc.) into electrical energy via the catalytic activity of electrogenic bacteria under anaerobic conditions [1,2,3]. They have attracted a lot of attention in scientific communities over the last decades not only as a solution for renewable power sources, but also as biosensors or water treatment devices [4,5,6,7]. Figure 1 exemplifies a double-chamber (anode and cathode) MFC with a Proton Exchange Membrane (PEM) separating the chambers.

In the last few years, researchers have mostly focused on the development of micro-scale MFCs (µMFCs) to employ them as portable devices [8,9]. Attributable to the advantages of micro-scale physics, µMFCs provide easy handling, higher surface areas, a lower consumption of substrates, quicker responses, and reduced distances for protons and electrons to travel [8,10,11,12]. In this scope, microelectromechanical systems (MEMS) technology is attractive for establishing µMFCs owing to the precise microfabrication techniques and economical mass production opportunities [10].

Macro-scale MFCs offer high power outputs, but a very long start-up time, which is the duration required to reach the maximum performance, and very high volumes ranging from mL to L, which cripple their practicality [6,11]. µMFCs have decreased this start-up time to a few days rather than weeks; however, researchers are yet to reach desired power outputs necessary for industrial applications. To exploit µMFCs as feasible power sources, their performance parameters, namely their power density, current density, and start-up time, must be further enhanced by decreasing their internal resistance [10,12].

The optimization of chamber and/or cell geometries, chamber or electrode materials, and electrode surface characteristics can be employed to decrease the internal resistance [6]. Optimizing the electrode surface characteristics can result in better surface contact for the bacterial biofilm. Biofilms are complex structures adhering to surfaces and consist of colonies of single bacterial species or multiple bacterial species. They can be found with a self-produced polymeric matrix attached to biotic or abiotic surfaces [13]. Their structure varies from Gram-positive to Gram-negative bacteria. The biofilm quality is affected not only by the interactions between the electrode surface and bacteria, but also by the operational parameters of the fuel cell [14,15]. Although bacterial adhesion to a surface is the first step of biofilm formation, other parameters also exist, such as hydrophobic interactions, electrostatic interactions, the electrode surface roughness, surface charges, and cell surface structures [16]. A study by Friman et al. published in 2013 showed that both the plankton bacterial cells and the biofilm contributed to the overall MFC voltage, and the biofilm contribution was three-times higher [17]. Additionally, Slate et al. reviewed studies showing the relation between the biofilm quality and power output [18].

Electrodes in fuel cells require a high conductivity, chemical stability, corrosion resistivity, and high mechanical strength. Since the anode is responsible for the growth of bacteria on the surface, it must have a good biocompatibility, as well as excellent electrical properties, to enable efficient electron transfer [19]. Generally, carbon-based materials are preferred in MFC studies due to their low cost. Another reason for their common use is that bacteria attach to carbon surfaces more easily, for instance, compared to bare gold surfaces. On the other hand, carbon-based materials have a major disadvantage, which is their low electrical conductivity, especially compared to gold. Although the cost of gold electrodes constricts their employment in large systems, they are widely used for fundamental research in miniaturized micro-scale MFC systems [20]. Gold surfaces can be enhanced by surface modification methods via the addition of different functional groups. Alkanethiols are widely preferred for modifying gold surfaces in the literature due to the strong and well-proven gold–thiol interaction [21]. Thiols form highly ordered self-assembled monolayers (SAMs) present on the gold surfaces. Since thiols with carboxyl or amine functional groups can bind to proteins on the bacterial membrane, the functionalization of gold surfaces with such groups will favor bacterial attachment [22,23] and this may, in turn, accelerate biofilm formation. However, these self-assembled monolayers may increase the electron transfer resistance, depending on the length of the carbon chain of the molecule to be used [24]. Cysteamine (CYS), an alkanethiol with an amine functional group, is known to favor bacterial attachment [25]. Another thiol is 11-mercaptoundecanoic acid (11-MUA), containing a carboxyl functional group. Some studies have shown that more current was generated by creating an SAM of carboxyl terminated alkanethiols compared to non-functionalized gold electrodes [22,26]. This was explained by the phenomenon in which carboxylic acid could accommodate cytochromes on the electrode surface through very strong hydrogen bonding with the peptide bonds in the protein backbone.

Our previous work reported MEMS-based double-chamber µMFC systems operated under different loads or an open circuit to compare the effect of different acclimatization conditions on the start-up time. Using *Shewanella oneidensis* MR-1 and bare gold anodes, the acclimatization of µMFCs under a finite load (25 kΩ) resulted in a shorter start-up time (30 h) compared to the µMFCs operated under an open circuit voltage (OCV). Power and current densities normalized to the anode area were 2 µW/cm^2^ and 12 µA/cm^2^, respectively [27]. In this study, the focus was placed on further decreasing the start-up time and internal resistance, while increasing the power output of MEMS-based µMFCs with a micro-liter volume. With this aim, surfaces of gold anode electrodes were modified with SAMs of aforementioned alkanethiol molecules (CYS and 11-MUA) and 4-aminothiophenol (4-ATP) to increase the biofilm quality favoring the bacterial attachment through functional groups on the surfaces. There are literature studies in which CYS and 11-MUA molecules have been used in MFCs [20,23]. 4-ATP, on the other hand, as far as the authors are aware, has not been studied in MFCs; however, the molecule has been widely employed in the functionalization of various surfaces, including conducting platforms and electrochemical applications [28,29], due to the fact that monolayers formed from aromatic thiols possess relatively high electrical conductivities [30].

## 2. Materials and Methods

### 2.1. Device Design, Fabrication, and Assembly

**Figure 2:**
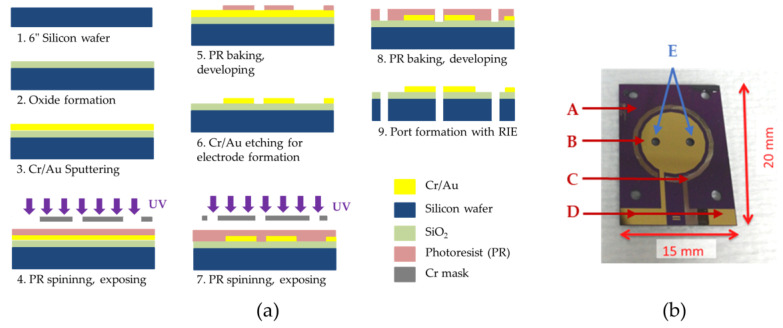
(**a**) Micro-scale Microbial Fuel Cell (MFC) (µMFC) electrode microfabrication steps; (**b**) microfabricated µMFC electrode. When two electrodes on substrates faced each other at 180°, the gold contact pads were exposed for electrical wiring. A: Silicon substrate; B: gold electrode; C: silver electrode (the brownish thin circular electrode around the gold electrode is the oxidized silver layer to be processed as the reference electrode for further studies); D: contact pads for electrical connection; and E: inlet and outlet ports.

Two-chamber MEMS-based µMFCs were designed to have circular gold electrodes of the same size in both the anode and cathode chamber. Through-holes were drilled through the silicon substrate for anolyte/catholyte feeding via microfluidic connection. The microfabrication masks used to define the gold electrodes were prepared with Cadence^®^ software (Virtuoso Layout Editor, Cadence Design Systems Inc., San Jose, CA, USA) and the microfabrication (Figure 2a) was performed in a class 1000 clean room area. Briefly, thermal oxide was formed on a 6″ polished silicon wafer by plasma-enhanced chemical vapor deposition (PECVD) to provide passivation. Cr/Au layers (30 nm/300 nm) were sputtered on the mentioned passivation layer and patterned by the standard photolithography process by coating photoresist (PR) on the wafer and exposing it with a mask under UV light. The through-holes were drilled via deep reactive-ion etching (DRIE). A picture of the microfabricated electrode is shown in Figure 2b.

**Figure 3:**
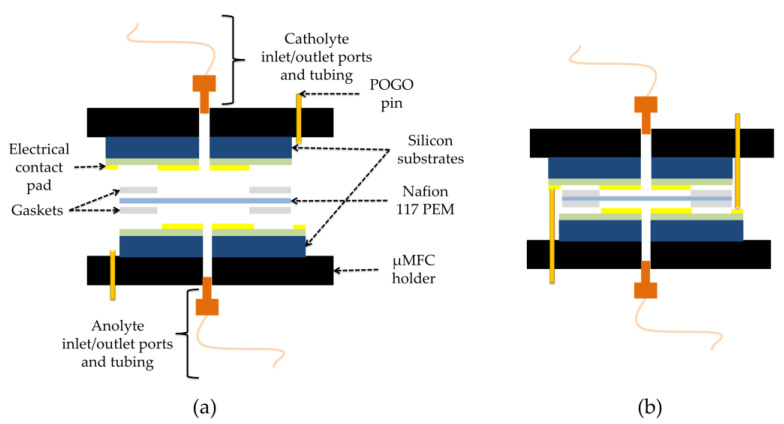
Schematic of µMFC: (**a**) extended view; (**b**) assembled view.

**Figure 4:**
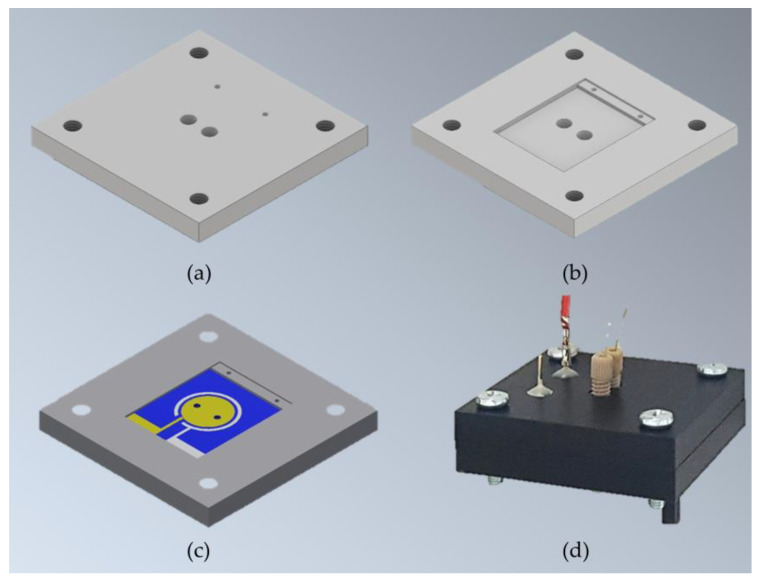
Schematic µMFC holder design and assembled µMFC: The holder is composed of two identical pieces of homoacetal polymer designed to house electrodes and permit fluidic and electrical connections. (**a**) Outer view of the holder piece; (**b**) inner view of the holder piece; (**c**) inner view of the holder piece; (**c**) holder inner view housing one of the electrodes; (**d**) assembled µMFC with microfluidic and electrical connections.

For the assembly, two pieces of Gel-Pak WF 1.5-X4 gel films (170 µm × 15 mm × 17 mm, with round holes at the center) were used as gaskets between the electrodes and proton exchange membrane (Nafion 117), which separates the anode and cathode chambers. All layers were manually stacked on top of each other (Figure 3) and tightly kept together inside the specially designed µMFC holder made of two pieces of an acetal homopolymer (Delrin^®^ by Dupont, Wilmington, DE, USA) (Figure 4).

Assembled µMFCs had two chambers (10.4 µL each), defined as anode and cathode chambers. The exposed conductive electrodes area per chamber was 0.61 cm^2^. The microfluidic inlet and outlet of the µMFC were connected from the outside of the silicon substrates via finger-tight PEEK (Polyether ether ketone) fittings (IDEX Health & Science) and capillary fused silica tubing (ID 150 µm, Postnova Analytics GmbH, Landsberg am Lech, Germany). Electrical connections were made by inserting pogo pins (spring-loaded electrically conductive pins) (OD 0.66 mm, FIXTEST GmbH, Engen, Germany) from the outside of the µMFC holder until the production of electrical contact pads of both anode and cathode electrodes. Figure 4 depicts the schematic holder design and an image of the assembled µMFC.

### 2.2. Surface Modification with Thiols

**Figure 5:**
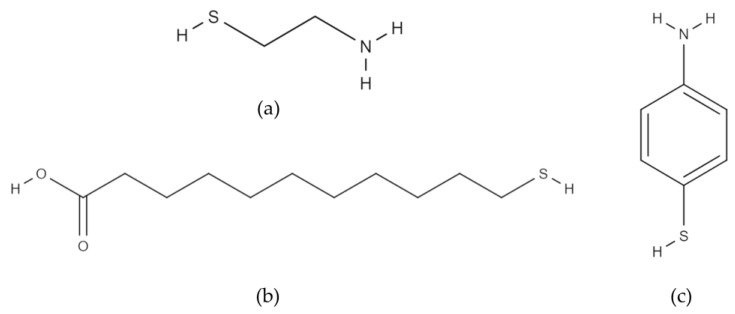
Thiol molecules used in the study: (**a**) cysteamine; (**b**) 11-mercaptoundecanoic acid; (**c**) 4-aminothiophenol.

To enhance the bacterial attachment on the surface, gold anode surfaces were modified with alkanethiols having different functional groups and chain lengths: amine terminated CYS and 4-ATP and carboxyl terminated 11-MUA (Sigma-Aldrich, St. Louis, MO, USA) (Figure 5).

The quality of the SAM on the gold surface is affected by several parameters, such as the gold surface cleanliness, solvent type, immersion time in solution, and concentration of the functional group. To obtain a good coverage on the gold surface, the procedure was completed as per the manufacturer’s instructions [31]. Protective photoresist layers on top of the gold electrodes were removed by immersion in acetone and IPA, respectively. Alkanethiol solutions with a 5 mM concentration were prepared separately in pure ethanol, as suggested by the manufacturer. For 11-MUA, the pH was adjusted to ∼2. For CYS and 4-ATP, the pH was adjusted to ∼12. The cleaned electrodes were immersed and incubated in either CYS, 11-MUA, or 4-ATP solutions for approximately 48 h. Later, the electrodes were rinsed with ethanol and dried with a stream of dry nitrogen gas [32,33]. The functionalized surfaces were characterized with contact angle measurement (KVS Attention Theta, METU Central Laboratory). The water contact angle was measured at room temperature using the sessile drop method by dropping 5 µL of water on both modified and unmodified surfaces. The contact angle was measured 5 s after the deposition of a drop by analyzing the image profile of the drop.

### 2.3. Inoculum

*Shewanella oneidensis* MR-1 (ATCC, Manassas, VA, USA) is a facultative anaerobic electrogenic bacterium. Glycerol stocks (−80 °C) of bacteria were grown on Tryptic Soy Agar (TSA, Merck) at 30 °C for 24 h by the streak plate method to obtain single colonies. Then, a single colony was cultured in Tryptic Soy Broth (TSB, Merck) medium on a shaker (150 rpm) at 30 °C for 24 h under aerobic conditions. Cell growth was monitored by colony-forming unit (CFU) determination (~2.5 × 10^6^ CFU/mL) by plating the cells onto TSA plates and incubating them aerobically at 30 °C. To be fed as the anolyte, fresh TSB and bacteria inoculum were mixed (1:1 v/v%). Throughout the operation, the inoculum ratio fed to the system was decreased and only pure TSB was fed after the third or fourth day.

### 2.4. MFC Operation

The anolyte (TSB mixed with inoculum at a volumetric ratio of 1:1 for the start-up) and catholyte solutions were continuously supplied using a syringe pump (KD Scientific) at rates of 3 μL/min and 5 μL/min, respectively, to anode and cathode chambers, independently. The catholyte was 100 mM potassium ferricyanide (K_3_[Fe(CN)_6_], Sigma-Aldrich) in a 100 mM phosphate buffer, in which the pH was adjusted at 7.5 ± 0.1. The µMFC was operated at 25 ± 1 °C. Different µMFC assemblies were connected to 25 kΩ external loads or operated under open circuit voltage conditions. The potential between the anode and cathode was measured via a digital multimeter (Agilent 3441A, Keysight Technologies) with a data acquisition system and recorded results every 1 min via a Keysight IntuiLink interface. All of the systems were operated under anaerobic conditions via the help of sealing gaskets between the anode and cathode chambers. The characteristics of µMFC systems operated in the study are presented in Table 1.

### 2.5. Performance Evaluation

The start-up time of the biofilm formation was determined using the voltage versus time plot, from the point when the voltage started to increase dramatically. The biofilm formation was accepted as having stopped when the voltage reached a steady value.

The performance of MFCs is usually estimated by a polarization curve. The polarization curve (voltage vs. current) was obtained by changing the external resistors between 1 MΩ and 0.5 kΩ at five-minute intervals while recording the voltage. Linear fitting of the curve in the ohmic loss region resulted in the total internal resistance of the µMFC. The current through the resistors was calculated via Ohm’s law, I = V/R, and the output power was obtained via Joule’s law, P = V × I. Current and power densities were normalized to the anode area (0.61 cm^2^) and anode chamber volume (10.4 µL).

The EIS measurements were performed with a two-electrode mode using the Autolab PGSTAT204 potentiostat/galvanostat FRA module (Metrohm) under open circuit conditions. Although the fabricated µMFC electrodes had silver layers to be used as the Ag/AgCl reference electrode after chlorination, this planar reference electrode was not suitable for long-term studies. Therefore, anode impedance spectra were obtained using the anode as the working electrode and the cathode functioning as both the reference and counter electrodes in a frequency range of 1 MHz to 0.1 Hz with a sinusoidal signal of a 10 mV amplitude. The data were fitted to an equivalent electrical circuit using the Autolab impedance analysis software Nova 1.11.

### 2.6. Biofilm Analysis

The anodes were disassembled and rinsed in phosphate buffer saline (PBS). Adherent bacteria on the anodes were fixed in a 2% glutaraldehyde solution for at least 24 h at 4 °C (glutaraldehyde solution, Grade I, 25% in H_2_O, Sigma Aldrich, St. Louis, MO, USA). Samples were then dehydrated by serial 10-min transfers through 50, 70, 90, and 100% ethanol. A thin Au-Pd film with a thickness of 3 nm was deposited onto the sample by sputtering to improve the conductivity for Scanning Electron Microscope (SEM) imaging. Prepared samples were examined at 20 kV using an SEM (FEI Quanta 400 F, METU Central Laboratory).

## 3. Results and Discussion

### 3.1. Characterization of Thiolated Gold Surfaces

Alkanethiol-modified gold surfaces having carboxylic or amine functional groups are known to result in a more hydrophilic character compared to bare gold surfaces. Table 2 shows the contact angle values for different surfaces. Carboxyl groups are amongst hydrophilic functional groups. Since the introduction of carboxylic acid on the surface led to an increase in the hydrophilicity, the water contact angle decreased approximately 11 degrees, which is in good agreement with the literature [34,35]. Functionalization with amine-terminated alkanethiols also led to a decrease in the water contact angle. Water molecules can interact with amine groups by forming hydrogen bonds, justifying the modification of the surface. The introduction of amine groups was also found to increase the hydrophilicity in other studies [36].

### 3.2. µMFC Performance

*Shewanella oneidensis* MR-1 used in the study can be simultaneously found as planktonic cells and adherent cells forming the biofilm in fuel cells, and both types can generate electricity alongside each other [37]. However, adherent cells on the anode are considered to be the main contributor to current generation in most cases [38]. Rozenfeld et al. showed that the biofilm on the anode provided the majority of the power output rather than planktonic bacteria in different micro electrolysis cells [39].

**Figure 6:**
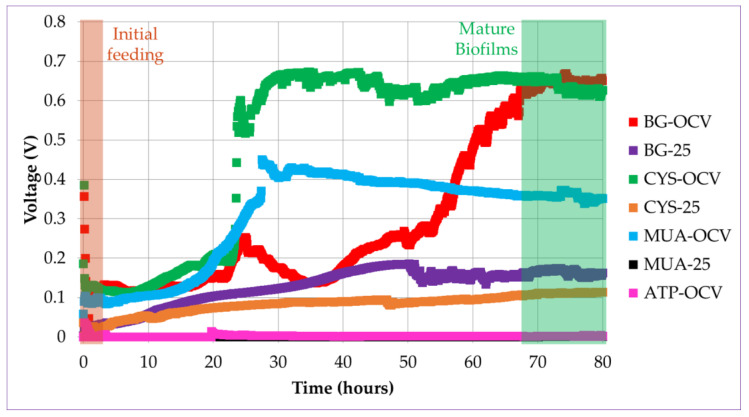
Voltage versus time plots for different µMFC systems during the start-up period (the voltage for the MUA-25 system was close to ATP-OCV).

In accordance with the mentioned literature, when introduced in the anode chamber, planktonic bacteria started oxidation of the fuel, causing a potential difference between the electrodes in this study. When the bacteria started to accumulate on the gold anode surface, the biofilm gradually formed. There was a rapid increase in voltage after some lag time, as shown in Figure 6. This lag time is associated with the time needed to establish initial bacteria-anode electron transfer. This increase was more apparent for the systems operated under OCV (BG-OCV, CYS-OCV, and MUA-OCV) than the systems operated under load (BG-25 and CYS-25). This can be observed in the voltage versus time plots of the µMFCs in Figure 6. CYS-25 and BG-25 system voltages started to increase after inoculation and kept increasing until reaching a maximum stable voltage value.

The time needed to reach 90% of the maximum stable voltage was determined as the start-up time. In our previous study, the acclimatization of µMFCs under a load resulted in a shorter start-up time [27]. In accordance with the previous results, in the study presented here, µMFCs with an unmodified anode (BG-25) resulted in a shorter start-up time when operated under load than BG-OCV. µMFCs functionalized with alkanethiols (CYS-OCV and MUA-OCV) displayed a greater than 50% shorter start-up time without the need of an operation under load with respect to the BG-OCV system. However, CYS-25 did not present advantages over CYS-OCV in terms of the start-up time. MUA-25 and ATP-OCV systems had very low voltages, so it was not possible to determine their start-up times and a quantitative performance analysis could not be performed (Table 3).

**Figure 7:**
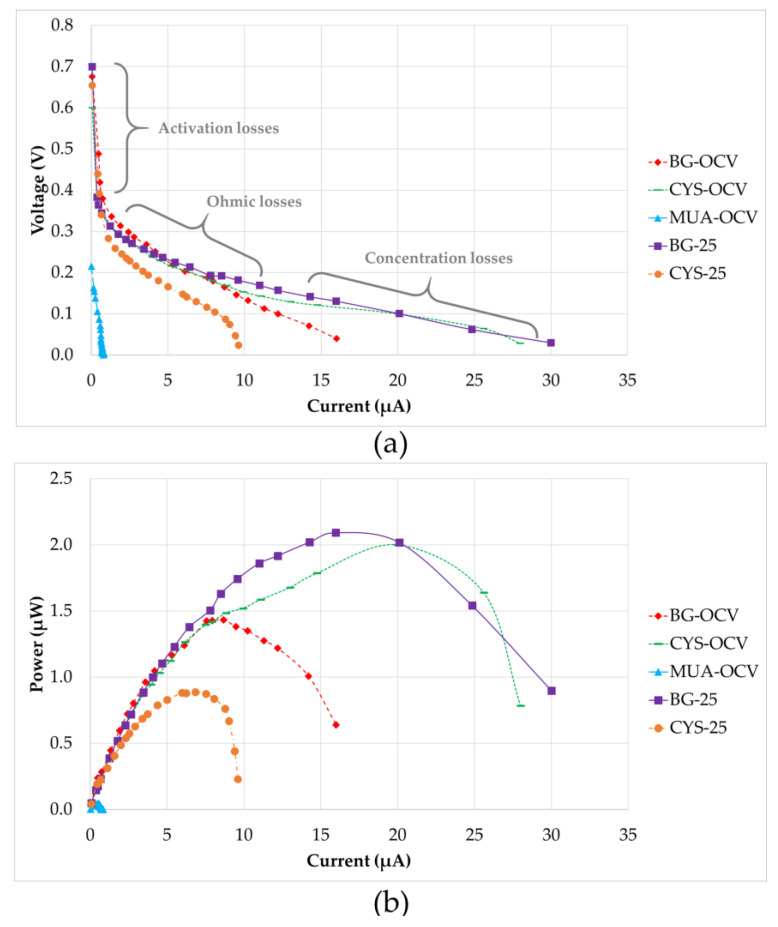
(**a**) Polarization curves (the regions are shown approximately) and (**b**) power output curves.

After the maturation of all systems, electrochemical analyses were performed. Figure 7 shows the polarization and power output curves of the five µMFCs investigated. The polarization plots consist of three regions. In the first region, activation losses were observed. This was characterized by a drastic decrease in voltage under a low current. As stated in the literature, these losses occur during the transfer of electrons at the electrode surface due to the activation energy needed for oxidation and reduction reactions [40]. In the second region, the voltages for all of the systems fell more slowly and the voltage drop had a linear relation with the current. This region is defined by the ohmic losses, which were both the resistance to the flow of electrons through the electrodes and interconnections, and the resistance to the flow of ions through anolyte, PEM, and catholyte. The slope of the linear part of the curve for ohmic losses defines the internal resistance value of the system. MUA-OCV had the highest internal resistance (214 kΩ), causing a very low power output. This can be explained by inefficient electron transfer through the anode and 11-MUA coating due to the long chain of this molecule. Internal resistances for CYS-25 (23 kΩ) and BG-OCV (19 kΩ) were in the same range, but BG-OCV had a higher power output (1.433 µW) because CYS-25 had not only a higher internal resistance, but also higher activation losses in the first region. As Figure 7b shows, the power outputs of BG-25 (2.093 µW) and CYS-OCV (2.000 µW) were close, but CYS-OCV had a 25% higher current value at the maximum power point due to a 33% lower internal resistance than BG-25. This demonstrated that CYS-OCV had a higher efficiency between the biofilm and anode surface, which may be due to better-quality biofilm formation. Concentration losses were dominant in the third region of the polarization curves, as can be observed in Figure 7. These losses occurred when the rate of mass transport of a chemical species by diffusion to the electrode surface inside the system limited the current production, mainly at high current densities, as stated by Logan et al. [40].

Table 3 provides a comparison of the quantitative results presented in Figure 7 for µMFCs investigated in this study. Although MUA-OCV had a shorter start-up time, its power output was very low. The bacteria quickly adhered to the modified surface and resulted in a shorter start-up time; however, since 11-MUA has a long carbon chain, its electron conductivity is lower than that of CYS, which affects the performance of the fuel cell. CYS-OCV was found to be quite promising because its power output was as high as BG-25 and it had a short start-up time. This can be explained by the favorable bacterial attachment on the amine group and rapid/uninhibited electron transfer due to the short carbon chain. On the other hand, ATP-OCV modified with 4-ATP did not generate power. Correspondingly, it is believed that phenol rings in 4-ATP might have inhibited bacterial growth, as some studies in the literature have claimed that phenols may have antimicrobial activity [41]. Furthermore, it was concluded that an increase in surface hydrophilicity had a positive effect on the power output in the case of the cysteamine-modified anode when BG-OCV and CYS-OCV were compared. Hydrophilicity makes surfaces more approachable for planktonic bacteria, as evidenced by [42,43], where an enhancement of the power output with an increase in the surface hydrophilicity was reported. However, it was observed in this study that hydrophilicity alone was not enough for this enhancement since modification with cysteamine and 3-ATP had a similar hydrophilicity, but different performance. It can be concluded that the functional group on the surface was also important, in addition to the hydrophilicity. 

The results obtained established certain performance enhancement in terms of power and current densities and the start-up time with respect to similar microliter-scale microbial fuel cells with the same biocatalyst used in the literature. The power density (315 µW/cm^3^) and start-up time (30 h) obtained with CYS-modified µMFCs (operated in an open circuit) were found to be better than in a similar literature study carried out by Qian et al. [7] (Table 4). The power and current densities in this study were smaller than the densities of MFCs made of carbon paper by Vigolo et al. [44], but the start-up time was five times shorter. Bacteria usually prefer to adhere to carbon-based materials, but they are difficult to integrate in MEMS processes compared to gold as an electrode material. There are studies being conducted to adapt carbon-based electrodes to micro-scale MFCs [45], but their fabrication is not yet compatible with mass production solutions.

### 3.3. EIS Characterization

Electrochemical Impedance Spectroscopy (EIS) is a technique widely used for the characterization of fuel cells to investigate electron transfer processes between interfaces. Its advantage over polarization curves is that EIS does not only give the total internal resistance of the fuel cell, but can also provide information about its components, such as the electrolyte resistance, charge transfer resistance, and diffusion resistance [46]. EIS forms the impedance spectra by applying an AC potential to an electrochemical cell and then measuring the current through this cell. 

**Figure 8:**
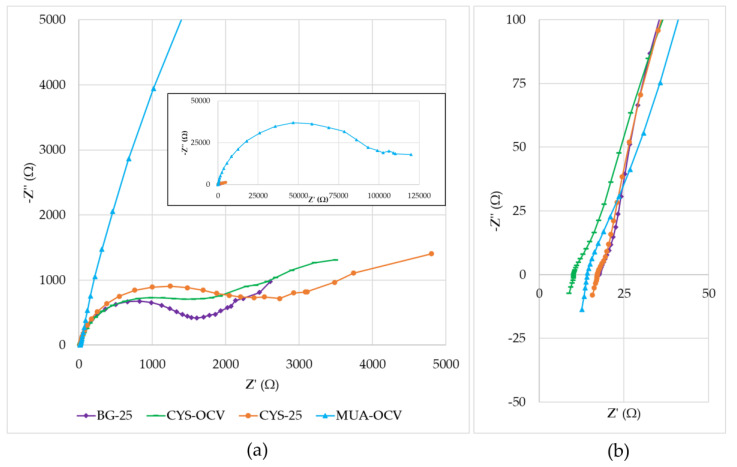
Nyquist plots: (**a**) Inset is the full view, including the low-frequency range for MUA-OCV; (**b**) close-up of the high-frequency region (1 MHz–5 kHz).

The µMFCs used in this study showed performance variations due to different resistances contained in their anode, cathode, electrolytes, and membrane. Figure 8 shows the Nyquist plots of overall fuel cells with a close-up view of the high-frequency region. When the Nyquist plots were compared, the primary observation was that the plots were formed by a distressed semi-circle (a characteristic of charge transfer process limitations) and a straight line in the low-frequency region, representing typical Warburg impedance (a characteristic of diffusion limitations). A distressed semi-circle is generally observed due to the combination of a capacitor element and a resistor element in parallel when fitting the EIS data. Determining the equivalent circuit of the system is crucial to interpreting these data. The Randles circuit is the most used equivalent circuit in the fitting of experimental data during EIS analysis. It is a model for a semi-infinite diffusion-controlled faradaic reaction to a planar electrode [47]. The diffusional resistance element (the Warburg impedance) is in series with the charge transfer resistance. It also includes the solution resistance and a double-layer capacitor.

By analyzing the data after fitting with the Randles circuit, it was observed that the main influence on the impedance was due to the charge transfer resistance. This was especially apparent with the very high impedance value (−Z” = 46.7 kΩ) of MUA-OCV. Its high impedance hindered the mass transfer effect in the low-frequency region. This supports the results obtained from polarization curves showing that MUA-OCV had inefficient charge transfer. Charge transfer resistances calculated using the fitted data for BG-25, CYS-OCV, CYS-25, and MUA-OCV were 1.3, 1.2, 1.8, and 60 kΩ. These values differed from the ones obtained using polarization curves. This difference may be corrected by using more accurate equivalent circuit models. Although the general behaviors of BG-25, CYS-OCV, and CYS-25 were similar, cysteamine-modified µMFCs had more prominent mass transfer limitations, as demonstrated by the low-frequency region in the Nyquist plots. These results were consistent with the study of Rikame et al. [48], where they concluded that the modified graphite anodes showed decreased charge transfer and electrolyte resistances due to active materials.

As demonstrated in Figure 8b, the solution resistance values changed between 10 and 20 Ω, which were much lower than the total internal resistance values. Additionally, the same plot showed that systems had non-zero inductances as its effects are often seen at the highest frequencies (between 1 and 0.15 MHz in the study). Possible causes are the actual physical inductance of the wires and the electrode. It points out that for developing a more accurate model, an inductor can also be included in the equivalent circuit.

### 3.4. Characterization of Biofilms

**Figure 9:**
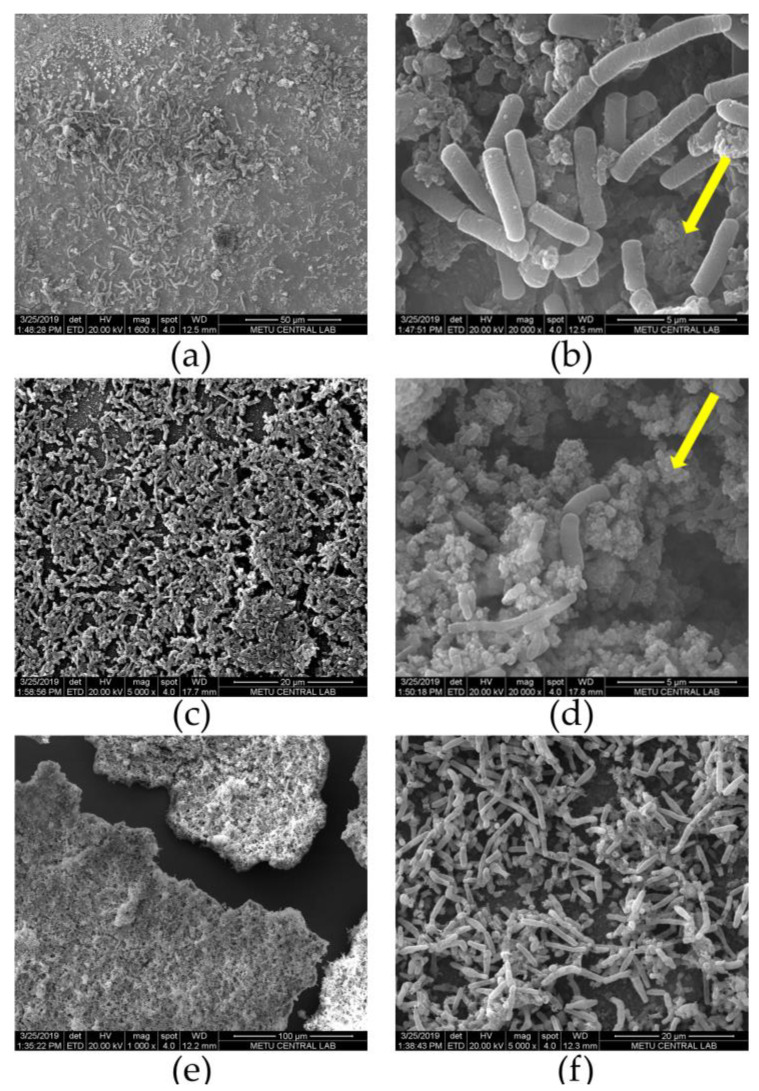
SEM images taken at 20 kV for different µMFC systems after the disassembly and fixation: (**a**,**b**) BG-25; (**c**,**d**) CYS-25; (**e**,**f**) MUA-OCV (arrows mark extracellular polymeric substances (EPS)).

After approximately two weeks of operation, µMFCs were disassembled and an SEM analysis of fixed gold anodes was performed. For three different µMFCs, SEM images showing *Shewanella oneidensis* MR-1 are given in Figure 9. CYS-25 and MUA-OCV systems resulted in very densely packed bacterial cells on the surface by stacking on top of each other. This was in good agreement with the relation between the start-up time and biofilm quality. Even though MUA-OCV produced a very low power output, it had a high-quality biofilm resulting in a shorter start-up time, which showed that 11-MUA does not inhibit biofilm growth, but electron transfer is inhibited by the long carbon chain of the molecule. The main difference between the appearances of biofilms was the amount of extracellular polymeric substances (EPS). MUA-OCV had less EPS and precipitated salts than CYS-25. The appearance of the bacteria was altered in some regions due to the fixation procedure. Furthermore, the treatment with ethanol might have washed away constituents, and dehydration might have affected the structure and thickness of the biofilm. There were visible regions where the biofilm was peeled off from the gold layer due to the damage that occurred during EIS measurements. When observed at a higher magnification (20,000×), there were voids and cracks on the biofilm. These structures help the mass transport by forming water channels. These cracks were also observed with the *Shewanella oneidensis* MR-1 biofilm grown on gold by Qian et al. [8] and on graphene foam by Jiang et al. [45]. These voids also lead to imperfect contact between the bacteria and the gold surface, causing a decrease in the electrical conductivity of the biofilm matrixes, which lowers the performance of the fuel cell, as suggested by other studies [49,50,51].

## 4. Conclusions

The influence of the surface functionalization of gold anodes with alkanethiols on the performance of microliter-scale MFCs employing an exoelectrogenic bacteria *Shewanella oneidensis* MR-1 was investigated. The experimental data showed that µMFCs modified with CYS and 11-MUA resulted in more than a 50% reduction in start-up times. Depending on the chain length and functional group of the molecule, power generation enhancement differed. SEM images confirmed that both 11-MUA and CYS modifications led to dense biofilms; however, their power outputs were lower than the output of bare gold µMFC acclimatized with a 25 kΩ load. The power and current densities achieved were comparable to those obtained in similar studies in the literature. These results provided insights into the interaction of electrogenic bacteria with different functional groups on gold surfaces. Further optimization studies with gold electrodes may enhance the performance of µMFCs for practical applications with a higher adaptability as fast and efficient portable devices, such as toxicity sensors, bacterial screening sensors, and micro power sources.

## Figures and Tables

**Table 1:** µMFC systems operated in the study.

System	Surface Treatment	Acclimatization Condition
BG-OCV	Bare gold	Under OCV conditions
BG-25	Bare gold	Under 25 kΩ load
CYS-OCV	Gold surface modified with Cysteamine	Under OCV conditions
CYS-25	Gold surface modified with Cysteamine	Under 25 kΩ load
MUA-OCV	Gold surface modified with 11-MUA	Under OCV conditions
MUA-25	Gold surface modified with 11-MUA	Under 25 kΩ load
ATP-OCV	Gold surface modified with 4-ATP	Under OCV conditions

**Table 2:** Contact angle measurements.

Surface	Contact Angle
Bare gold	65°
Gold surface modified with cysteamine	52°
Gold surface modified with 11-MUA	53.7°
Gold surface modified with 4-ATP	52°

**Table 3:** Performance µMFC systems.

Parameter	BG-OCV	BG-25	CYS-OCV	CYS-25	MUA-OCV
Load during operation	OCV	25 kΩ	OCV	25 kΩ	OCV
Start-up time	70 h	48 h	30 h	42 h	27 h
Internal resistance	19 kΩ	8 kΩ	6 kΩ	23 kΩ	214 kΩ
Maximum power	1.433 µW	2.093 µW	2.000 µW	0.889 µW	0.046 µW
Current at max. power	8.684 µA	15.976 µA	20.000 µA	6.842 µA	0.527 µA

**Table 4:** Comparison of µMFC systems with the literature.

Parameter	BG-25	CYS-OCV	Qian et al., 2009 [7]	Vigolo et al., 2014 [44]
Bacteria	*S. oneidensis* MR-1	*S. oneidensis* MR-1	*S. oneidensis* MR-1	*S. oneidensis* MR-1
Fuel cell geometry	Double chamber with Nafion-117	Double chamber with Nafion-117	Double chamber with Nafion-117	Double chamber with Nafion-117
Process type	Continuous	Continuous	Batch	Continuous
Anode area	0.61 cm^2^	0.61 cm^2^	0.15 cm^2^	0.5 cm^2^
Anode volume	10.4 µL	10.4 µL	5 µL	5 µL
Chamber depth	170 µm	170 µm	100 µm	100 µm
Anode/Cathode materials	Au/Au	Au/Au	Au/Carbon cloth	Carbon paper/Carbon paper
Anolyte/Catholyte	TSB/K_3_Fe(CN)_6_	TSB/K_3_Fe(CN)	TSB/K_3_Fe(CN)	TSB/K_3_Fe(CN)
Load	25 kΩ	OCV	100 Ω	100 kΩ
Start-up time	48 h	30 h	47 h	160 h(15 h lag time)
Internal resistance	8 kΩ	6 kΩ	30 kΩ	49 kΩ
Volumetric power density	330 µW/cm^3^	315 µW/cm^3^	15 µW/cm^3^	900 µW/cm^3^
Areal power density	3.4 µW/cm^2^	3.3 µW/cm^2^	0.15 µW/cm^2^	9 µW/cm^2^
Volumetric current density	2515 µA/cm^3^	3148 µA/cm^3^	1300 µA/cm^3^	6200 µA/cm^3^
Areal current density	26.1 µA/cm^2^	32.7 µA/cm^2^	13 µA/cm^2^	62 µA/cm^2^

## References

[1] Du Z., Li H., Gu T. (2007). A State of the Art Review on Microbial Fuel Cells: A Promising Technology for Wastewater Treatment and Bioenergy. Biotechnol. Adv..

[2] Lovley D.R. (2006). Bug Juice: Harvesting Electricity with Microorganisms. Nat. Rev. Microbiol..

[3] Rabaey K., Verstraete W. (2005). Microbial Fuel Cells: Novel Biotechnology for Energy Generation. Trends Biotechnol..

[4] Bond D.R., Lovley D.R. (2003). Electricity Production by Geobacter Sulfurreducens Attached to Electrodes. Appl. Environ. Microbiol..

[5] Dávila D., Esquivel J.P., Sabaté N., Masa J. (2011). Silicon-based microfabricated microbial fuel cell toxicity sensor. Biosens. Bioelectron..

[6] Choi S. (2015). Microscale microbial fuel cells: Advances and challenges. Biosens. Bioelectron..

[7] Cui Y., Lai B., Tang X. (2019). Microbial Fuel Cell-Based Biosensors. Biosensors.

[8] Qian F., Baum M., Gu Q., Morse D.E. (2009). A 1.5 µL microbial fuel cell for on-chip bioelectricity generation. Lab Chip.

[9] Choi S., Lee H.-S., Yang Y., Parameswaran P., Torres C.I., Rittmann B.E., Chae J. (2011). A μL-scale Micromachined Microbial Fuel Cell Having High Power Density. Lab Chip.

[10] Ren H., Lee H.-S., Chae J. (2012). Miniaturizing microbial fuel cells for potential portable power sources: Promises and challenges. Microfluid. Nanofluidics.

[11] Wang H.Y., Bernarda A., Huang C.Y., Lee D.J., Chang J.S. (2011). Micro-sized microbial fuel cell: A mini-review. Bioresour. Technol..

[12] Choi S., Chae J. (2013). Optimal biofilm formation and power generation in a micro-sized microbial fuel cell (MFC). Sens. Actuators A Phys..

[13] Kumar R., Singh L., Wahid Z.A., Din M.F.M. (2015). Exoelectrogens in microbial fuel cells toward bioelectricity generation: A review. Int. J. Energy Res..

[14] Read S.T., Dutta P., Bond P.L., Keller J., Rabaey K. (2010). Initial Development and Structure of Biofilms on Microbial Fuel Cell Anodes. BMC Microbiol..

[15] Kumar R., Singh L., Zularisam A.W. (2016). Exoelectrogens: Recent advances in molecular drivers involved in extracellular electron transfer and strategies used to improve it for microbial fuel cell applications. Renew. Sustain. Energy Rev..

[16] Angelaalincy M.J., Krishnaraj R.N., Shakambari G., Ashokkumar B., Kathiresan S., Varalakshmi P. (2018). Biofilm Engineering Approaches for Improving the Performance of Microbial Fuel Cells and Bioelectrochemical Systems. Front. Energy Res..

[17] Friman H., Schechter A., Ioffe Y., Nitzan Y., Cahan R. (2013). Current production in a microbial fuel cell using a pure culture of C upriavidus basilensis growing in acetate or phenol as a carbon source. Microb. Biotechnol..

[18] Slate A.J., Whitehead K.A., Brownson D.A.C., Banks C.E. (2019). Microbial fuel cells: An overview of current technology. Renew. Sustain. Energy Rev..

[19] Mink J.E., Qaisi R.M., Logan B.E., Hussain M.M. (2014). Energy harvesting from organic liquids in micro-sized microbial fuel cells. NPG Asia Mater..

[20] Baudler A., Schmidt I., Langner M., Greiner A., Schröder U. (2015). Does it have to be carbon? Metal anodes in microbial fuel cells and related bioelectrochemical systems†. Energy Environ. Sci..

[21] Xue Y., Li X., Li H., Zhang W. (2014). Quantifying thiol–gold interactions towards the efficient strength control. Nat. Commun..

[22] Crittenden S.R., Sund C.J., Sumner J.J. (2006). Mediating Electron Transfer from Bacteria to a Gold Electrode via a Self-Assembled Monolayer. Langmuir.

[23] Guo K., Freguia S., Dennis P.G., Chen X., Donose B.C., Keller J., Gooding J.J., Rabaey K. (2013). Effects of Surface Charge and Hydrophobicity on Anodic Biofilm Formation, Community Composition, and Current Generation in Bioelectrochemical Systems. Environ. Sci. Technol..

[24] Füeg M., Borjas Z., Estevez-Canales M., Esteve-Núñez A., Pobelov I.V., Broekmann P., Kuzume A. (2019). Interfacial electron transfer between Geobacter sulfurreducens and gold electrodes via carboxylate-alkanethiol linkers: Effects of the linker length. Bioelectrochemistry.

[25] La J.A., Jeon J.-M., Sang B.-I., Yang Y.-H., Cho E.C. (2017). A Hierarchically Modified Graphite Cathode with Au Nanoislands, Cysteamine, and Au Nanocolloids for Increased Electricity-Assisted Production of Isobutanol by Engineered Shewanella oneidensis MR-1. Appl. Mater. Interfaces.

[26] Lowy D.A., Tender L.M., Zeikus J.G., Park D.H., Lovley D.R. (2006). Harvesting Energy From the Marine Sediment-Water Interface II. Kinetic Activity of Anode Materials. Biosens. Bioelectron..

[27] Şen Doğan B., Afşar Erkal N., Özgür E., Zorlu Ö., Külah H. (2016). Performance Enhancement of Mems-Based Microbial Fuel Cells (μMFC) For Microscale Power Generation. J. Phys. Conf. Ser..

[28] Khan A., Jawaid M., Neppolian B., Asiri A.M. (2019). Graphene Functionalization and Nanopolymers. Graphene Functionalization Strategies: From Synthesis to Applications.

[29] Abaci S., Shannon C. (2005). The influence of decanethiol/4-aminothiophenol mixed monolayers on the electrodeposition of polyaniline thin films. Electrochim. Acta.

[30] Hayes W.A., Shannon C. (1996). Electrochemistry of Surface-Confined Mixed Monolayers of 4-Aminothiophenol and Thiophenol on Au. Langmuir.

[31] Sigma-Aldrich Technical Bulletin AL-266. https://www.sigmaaldrich.com/content/dam/sigma-aldrich/docs/Aldrich/Instructions/1/al-266.pdf?utm_source=redirect&utm_medium=promotional&utm_campaign=insite_al_techbull_al266.

[32] Shen H., Mark J.E., Seliskar C.J., Mark H.B., Heineman W.R. (1997). Blocking behavior of self-assembled monolayers on gold electrodes. J. Solid State Electrochem..

[33] Ashaduzzaman M., Antony A.A., Murugan N.A., Deshpande S.R., Turner A., Tiwari A. (2015). Studies on an on/off-switchable immunosensor for troponin T. Biosens. Bioelectron..

[34] Afara N., Omanovic S., Asghari-Khiavi M. (2012). Functionalization of a gold surface with fibronectin (FN) covalently bound to mixed alkanethiol self-assembled monolayers (SAMs): The influence of SAM composition on its physicochemical properties and FN surface secondary structure. Thin Solid Films.

[35] Veiseh M., Zareie M.H., Zhang M. (2002). Highly selective protein patterning on gold-silicon substrates for biosensor applications. Langmuir.

[36] Dharanivasan G., Rajamuthuramalingam T., Jesse D.M.I., Rajendiran N., Kathiravan K. (2015). Gold nanoparticles assisted characterization of amine functionalized polystyrene multiwell plate and glass slide surfaces. Appl. Nanosci..

[37] Borole A.P., Reguera G., Ringeisen B., Wang Z.-W., Feng Y., Kim B.H. (2011). Electroactive biofilms: Current status and future research needs. Energy Environ. Sci..

[38] Ringeisen B.R., Hendeson E., Wu P.K., Pietron J., Ray R., Little B., Biffinger J.C., Jones-Meehan J.M. (2006). High power density from a miniature microbial fuel cell using Shewanella oneidensis DSP10. Environ. Sci. Technol..

[39] Rozenfeld S., Hirsch L.O., Gandu B., Farber R., Schechter A., Cahan R. (2019). Improvement of Microbial Electrolysis Cell Activity by Using Anode Based on Combined Plasma-Pretreated Carbon Cloth and Stainless Steel. Energies.

[40] Logan B.E., Hamelers B., Rozendal R., Schröder U., Keller J., Freguia S., Aelterman P., Verstraete W., Rabaey K. (2006). Microbial Fuel Cells: Methodology and Technology†. Environ. Sci. Technol..

[41] Walsh D.J., Livinghouse T., Goeres D.M., Mettler M., Stewart P.S. (2019). Antimicrobial Activity of Naturally Occurring Phenols and Derivatives Against Biofilm and Planktonic Bacteria. Front. Chem..

[42] Jia Y., Feng H., Shen D., Zhou Y., Chen T., Wang M., Chen W., Ge Z., Huang L., Zheng S. (2018). High-performance microbial fuel cell anodes obtained from sewage sludge mixed with fly ash. J. Hazard. Mater..

[43] Du Q., An J., Li J., Zhou L., Li N., Wang X. (2017). Polydopamine as a new modification material to accelerate startup and promote anode performance in microbial fuel cells. J. Power Sources.

[44] Vigolo D., Al-Housseiny T.T., Shen Y., Akinlawon F.O., Al-Housseiny S.T., Hobson R.K., Sahu A., Bedkowski K.I., DiChristina T.J., Stone H.A. (2014). Flow dependent performance of microfluidic microbial fuel cells†. Phys. Chem. Chem. Phys..

[45] Jiang H., Ali M.A., Xu Z., Halverson L.J., Dong L. (2017). Integrated Microfluidic Flow-Through Microbial Fuel Cells. Sci. Rep..

[46] Aelterman P., Rabaey K., Pham H.T., Boon N., Verstraete W. (2006). Continuous Electricity Generation at High Voltages and Currents Using Stacked Microbial Fuel Cells. Environ. Sci. Technol..

[47] Randviir E.P., Banks C.E. (2013). Electrochemical impedance spectroscopy: An overview of bioanalytical applications. Anal. Methods.

[48] Rikame S.S., Mungray A.A., Mungray A.K. (2018). Modification of anode electrode in microbial fuel cell for electrochemical recovery of energy and copper metal. Electrochim. Acta.

[49] Torres C.I., Kato Marcus A., Rittmann B. (2008). Proton transport inside the biofilm limits electrical current generation by anode-respiring bacteria. Biotechnol. Bioeng..

[50] Zhang L., Zhu X., Li J., Liao Q., Ye D. (2011). Biofilm formation and electricity generation of a microbial fuel cell started up under different external resistances. J. Power Sources.

[51] Li J., Li H., Zheng J., Zhang L., Fu Q., Zhu X., Liao Q. (2017). Response of anodic biofilm and the performance of microbial fuel cells to different discharging current densities. Bioresour. Technol..

